# Normalisation of High Bone Remodelling due to Oestrogen Deficiency by Traditional Chinese Formulation *Kang Shuai Lao Pian* in Ovariectomised Rats

**DOI:** 10.7150/ijms.75915

**Published:** 2022-09-21

**Authors:** Sophia Ogechi Ekeuku, Kok-Yong Chin, Jing Qian, Haibin Qu, Yi Wang, Elvy Suhana Mohd Ramli, Sok Kuan Wong, Mohd Mustazil Mohd Noor, Soelaiman Ima-Nirwana

**Affiliations:** 1 Department of Pharmacology, Faculty of Medicine, Universiti Kebangsaan Malaysia, Kuala Lumpur, Malaysia.; 2 Pharmaceutical Informatics Institute, College of Pharmaceutical Sciences, Zhejiang University, Hangzhou, China.; 3 Department of Anatomy, Faculty of Medicine, Universiti Kebangsaan Malaysia, Kuala Lumpur, Malaysia.

**Keywords:** Traditional Chinese medicine, skeletal health, postmenopausal osteoporosis

## Abstract

Postmenopausal osteoporosis transpires due to excessive osteoclastic bone resorption and insufficient osteoblastic bone formation in the presence of oestrogen insufficiency. *Kang Shuai Lao Pian* (KSLP) is a red ginseng-based traditional Chinese medicine known for its anti-ageing properties. However, studies on its effect on bone loss are lacking. Thus, the current study examined the skeletal protective effects of KSLP in an ovariectomised rodent bone loss model. Three-month-old female Sprague Dawley rats (n=42) were randomised into baseline, sham and ovariectomised (OVX) groups. The OVX rats were supplemented with low- (KSLP-L; 0.15 g/kg), medium- (KSLP-M; 0.30 g/kg), high-dose KSLP (KSLP-H; 0.45 g/kg) or calcium carbonate (1% w/v). The daily supplementation of KSLP was performed via oral gavage for eight weeks. Gavage stress was stimulated in the ovariectomised control with distilled water. The rats were euthanised at the end of the study. Whole-body and femoral bone mineral content and density scans were performed at baseline and every four weeks. Blood samples were obtained for the determination of bone remodelling markers. Histomorphometry and biomechanical strength testing were performed on femurs and tibias. High bone remodelling typically due to oestrogen deficiency, indicated by the elevated bone formation and resorption markers, osteoclast surface, single-labelled surface and mineralising surface/bone surface ratio, was observed in the untreated OVX rats. Whole-body BMD adjusted to body weight and Young's modulus decreased significantly in the untreated OVX rats. High-dose KSLP supplementation counteracted these degenerative changes. In conclusion, KSLP improves bone health by normalising bone remodelling, thereby preventing bone loss and decreased bone strength caused by oestrogen deficiency. Its anti-osteoporosis effects should be validated in patients with postmenopausal osteoporosis.

## Introduction

The most prevalent type of osteoporosis among women is postmenopausal osteoporosis 1. Oestrogen deficiency due to the cessation of ovarian function results in increased bone resorption mediated by osteoclasts, followed by compensatory bone formation by osteoblasts. However, the bone resorption rate outpaces the bone formation rate, producing a net bone loss 2. Fragility fractures due to postmenopausal osteoporosis contribute significantly to morbidity and mortality globally 3.

Oral bisphosphonates are the first-line treatment for primary osteoporosis. Bisphosphonates attach to hydroxyapatite and suppress osteoclastic bone resorption. In the event of intolerance or ineffectiveness, bisphosphonates may be replaced with intravenous bisphosphonates, strontium ranelate, denosumab (monoclonal anti-receptor activator of nuclear factor kappa-B ligand antibody), teriparatide (parathyroid hormone fragment 1-34), abaloparatide (parathyroid hormone-related protein analogue) or romosozumab (monoclonal anti-sclerostin antibody). Raloxifene and hormone replacement are additional osteoporosis treatment options for postmenopausal women 4. These drugs come with undesirable side effects despite their proven efficacy in improving bone mineral density (BMD) and preventing fractures. Calcium and vitamin D remain the current preventive agents for osteoporosis 5.

For centuries, traditional Chinese medicine (TCM) has been used to manage various orthopaedic conditions, such as bone fractures, rheumatism and osteoporosis 6-8. *Kang Shuai Lao Pian* (KSLP) is a red ginseng-based TCM formulation believed to be inherited from a Ming Dynasty court prescription 9. It has been marketed as an anti-ageing product in China. The ingredients of KSLP include *Rehmannia glutinosa* (Gaertn.) DC., *Panax ginseng* C.A.Mey., *Asparagus cochinchinensis* (Lour.) Merr., *Ophiopogon japonicus* (Thunb.) Ker Gawl., *Lycium chinense* Mill., and *Poria cocos* (Schw.) Wolf. 9. Each of these ingredients has been previously demonstrated to protect against osteoporosis and/or improve oxidative stress in preclinical studies 10-15. A preliminary study has suggested that KSLP promoted osteogenic differentiation of adipose-derived stem cells in aged mice 16. However, the skeletal effects of KSLP have not been validated in an animal model of osteoporosis. Therefore, the current study attempts to examine the skeletal protective effects of KSLP in an ovariectomized rat model of osteoporosis.

## Material and methods

### Preparation of treatment

Chiatai Qingchunbao Pharmaceutical Co. Ltd. (Hangzhou, China) provided the KSLP (Med-drug permit no. B20021021) used in the study. The six herbs used, i.e. *R. glutinosa* (Gaertn.) DC., *P. ginseng* C.A.Mey., *A. cochinchinensis* (Lour.) Merr., *O. japonicus* (Thunb.) Ker Gawl., *L. chinense* Mill., and *P. cocos* (Schw.) Wolf., were mixed at the ratio 409:167:26:26:26:77 by weight. The preparation of KSLP has been as described in the patent CN 1943707 B. Data on the high-performance liquid chromatography fingerprint of KSLP were available in the study of Gong et al. 9.

The recommended human dose of KSLP as per the product sheet is 3.4 g/day, or 0.057 g/kg/day for a 60-kg human adult. Based on the body surface ratio conversion formula 17, the animal dose was estimated to be 0.3 g/kg for a 400-g rat (body weight at 4 months old). Three different doses of KSLP were used in this study, i.e. 0.15 (low), 0.3 (medium), and 0.45 g/kg/day to demonstrate a dose-dependent effect. KSLP was dissolved in distilled water before oral administration.

### Animals

The Laboratory Animal Resource Unit, Universiti Kebangsaan Malaysia (Kuala Lumpur, Malaysia) provided 42 three-month-old female Sprague-Dawley rats used in this study. They were housed at the animal laboratory of the Pharmacology Department at Universiti Kebangsaan Malaysia under standard conditions (8:16 light/dark cycle, 27±1°C ambient temperature). The rats were provided with standard rat chow (702P, Gold Coin, Klang, Malaysia) and tap water ad libitum. The Universiti Kebangsaan Malaysia Animal Ethics Committee reviewed and approved the study (approval code: FAR/FP/KOK YONG/25-MAR./1088-MAR.-2020-FEB.-2022). The handling of animals was performed in accordance with the Animal Welfare Act 2015 of Malaysia.

### Study design

After a seven-day acclimatisation period, the rats were randomised into three groups: baseline (n=6), sham-operated (n=6), and ovariectomized (OVX) (n=30). The OVX rats were further assigned into five groups, i.e. OVX control, OVX supplemented with low- (KLSP-L), medium- (KLSP-M), high-dose (KLSP-H) KSLP or calcium carbonate (1% CaCO_3_ in drinking water). The baseline group was sacrificed immediately without any intervention. All other groups underwent bilateral ovariectomy to induce oestrogen deficiency, except the sham group which underwent laparotomy but their ovaries were not removed. Seven days after ovariectomy, the sham, OVX control and OVX rats supplemented with 1% CaCO_3_ were given distilled water via oral gavage to stimulate gavage stress, while the KSLP-supplemented groups received KSLP solution at 0.15, 0.30 and 0.45 g/kg/day via oral gavage daily for eight weeks. The OVX rats were given 1% CaCO_3_ (w/v) in their drinking water (ad libitum). The body weight of the rats was monitored weekly using an electronic weighing scale (Comanche CMKS-690-6kg). At the start of treatment, one and two months later, whole-body and regional bone mineral density (BMD) and content (BMC) of the rats were measured. Subcutaneous injection of calcein (10 mg/mL) was given nine and two days before sacrifice for dynamic histomorphometry. Blood samples were taken before and after the eight-week treatment, centrifuged at 3000 rpm for 10 minutes to extract the serum, which was then stored at -70 °C until analysis. At the end of the eight-week treatment, the tibia and femur samples of the rats were harvested. The left femur was stored in 10% neutral buffered formalin while the rest of the bone samples were stored at -80 °C.

### Dual-energy X-ray absorptiometry (DXA) analysis

The anaesthetised rats were placed ventrally (0.1 mL/100 g ketamine/xylazil/zoletil mixture). Whole-body BMD, fat mass and fat percentage were obtained from a whole-body DXA scan, while femoral BMD and BMC were obtained from a high-resolution scan. All scans were conducted with Discovery Wi Bone Densitometer (Hologic, MA, USA) with small animal analysis software. The short-term coefficient of variation for this machine was 1.4% for whole-body BMD in rats 18.

### Bone remodelling markers

Commercial enzyme-linked immunoassay kits were used to measure serum osteocalcin (bone formation marker; QY-E11635, Qayee-Bio, Shanghai, China) and carboxy-terminal cross-linked telopeptide of type 1 collagen (CTX-1) (bone resorption marker; QY-E11374, Qayee-Bio, Shanghai, China) levels per the manufacturer's instructions. Pre- and post-treatment serum samples were analysed.

### Bone histomorphometry

The left femur cleaned of soft tissue was sawed in halves. Half of the femur was decalcified in 10% ethylenediaminetetraacetic acid (EDTA) for 30 days, embedded in paraffin wax, and processed into 5-μm thick sections with a microtome (Leica RM2235, Nussloch, Germany). The slides were rinsed with xylene to eliminate the paraffin and rehydrated. They were stained with haematoxylin for 10 minutes and eosin for 5 minutes. Some of the slides were stained with tartrate-resistance acid phosphatase (TRAP) solution [mixture of TRAP basic incubation medium (sodium acetate anhydrous (Chemiz, Malaysia), L-(+) tartaric acid (Sigma-Aldrich, USA), glacial acetic acid (Merck, Germany)), fast red violet LB salt (Sigma-Aldrich, USA)) and naphthol AS-MX phosphate substrate mix (naphthol AS-MX phosphate (Sigma-Aldrich, USA) and diethylene glycol diethyl ether (Sigma-Aldrich, USA))] and incubated at 37^o^C until the sections turn yellowish. The TRAP-stained slides were counter-stained with 0.08% fast green for 59 mins. After dehydration and the subsequent mounting, all slides were examined using a light microscope (Zeiss Primo Star, Germany) at a magnification of 100 × with the aid of Zen 2.6 lite software. For bone cellular parameters, osteoblast surface (Ob.S/BS), eroded surface (ES/BS), osteoid surface (OS/BS), osteoid volume (OV/BV) and osteoclast surface (Oc.S/BS) were determined.

The undecalcified half of the femur was embedded in polymethyl methacrylate (Polysciences, PA, USA) and sectioned with a microtome (Leica RM2235, Nussloch, Germany) at 8 μm thickness. The slides were examined under a fluorescence microscope (Olympus BX53, Japan) at a magnification of 200 × with the aid of Olympus cellSens standard 1.14 software. The bone dynamic parameters assessed using the unstained undecalcified sections were single-labelled surface (sLS/BS), doubled-labelled surface (dLS/BS), mineralising surface (MS/BS), mineral apposition rate (MAR), and bone formation rate (BFR).

Some of the undecalcified sections were soaked in acetone, rehydrated, flooded with 1% silver nitrate under ultraviolet light for 20 mins, rinsed with distilled water and flooded with 2.5% thiosulfate solution for 5 mins. Stained slides were then dehydrated in increasing strengths of alcohol and cleared with diethyl ether. The slides were examined under a light microscope (Zeiss Primo Star, Germany) and images were captured at a magnification of 100 × with the Zen 2.6 lite software. The structural bone indices assessed were bone volume/total volume (BV/TV), trabecular bone thickness (Tb.Th), trabecular bone number (Tb.N), and trabecular bone separation (Tb.Sp).

Cellular and dynamic parameters of the femoral metaphysis were obtained using the Weibel Grid technique.

### Biomechanical strength

The frozen femurs were thawed at room temperature. They were weighed and measured in length and width. A three-point bending test was performed on the mid-diaphysis of the left femur using Shimadzu Universal Testing Machine (Autograph AGS-X 500N, Japan). A load was applied onto each bone at the midpoint between two lower supports (10-mm span) with the anterior aspect facing down at a speed of 5 mm/min till fracture. The Trapezium X software was used to generate the load (N), stress (N/mm^2^), displacement (mm) and strain (%) data of the bone. The stiffness was calculated by dividing the load (N) by the displacement (mm), and Young's Modulus of elasticity was calculated by dividing the stress (N/mm^2^) by the strain (%).

### Statistical analysis

Statistical analysis was performed using Statistical Package for Social Sciences version 23.0 (IBM, Armonk, USA). Shapiro-Wilk test was used to determine the normality of the data. One-way analysis of variance (ANOVA) with Tukey's post hoc test was used to compare the mean difference among the study groups. Mixed-design ANOVA with small effect analysis was used to analyse time and treatment effects. The data were presented as mean ± standard error of the mean (SEM). Skewed data were compared using Kruskal Wallis test and Mann Whitney U-test with Bonferroni adjustment. They were presented as the median and interquartile range (IQR). A p-value of <0.05 was considered statistically significant.

## Results

### Body weight and body composition analysis

**Figure 1:**
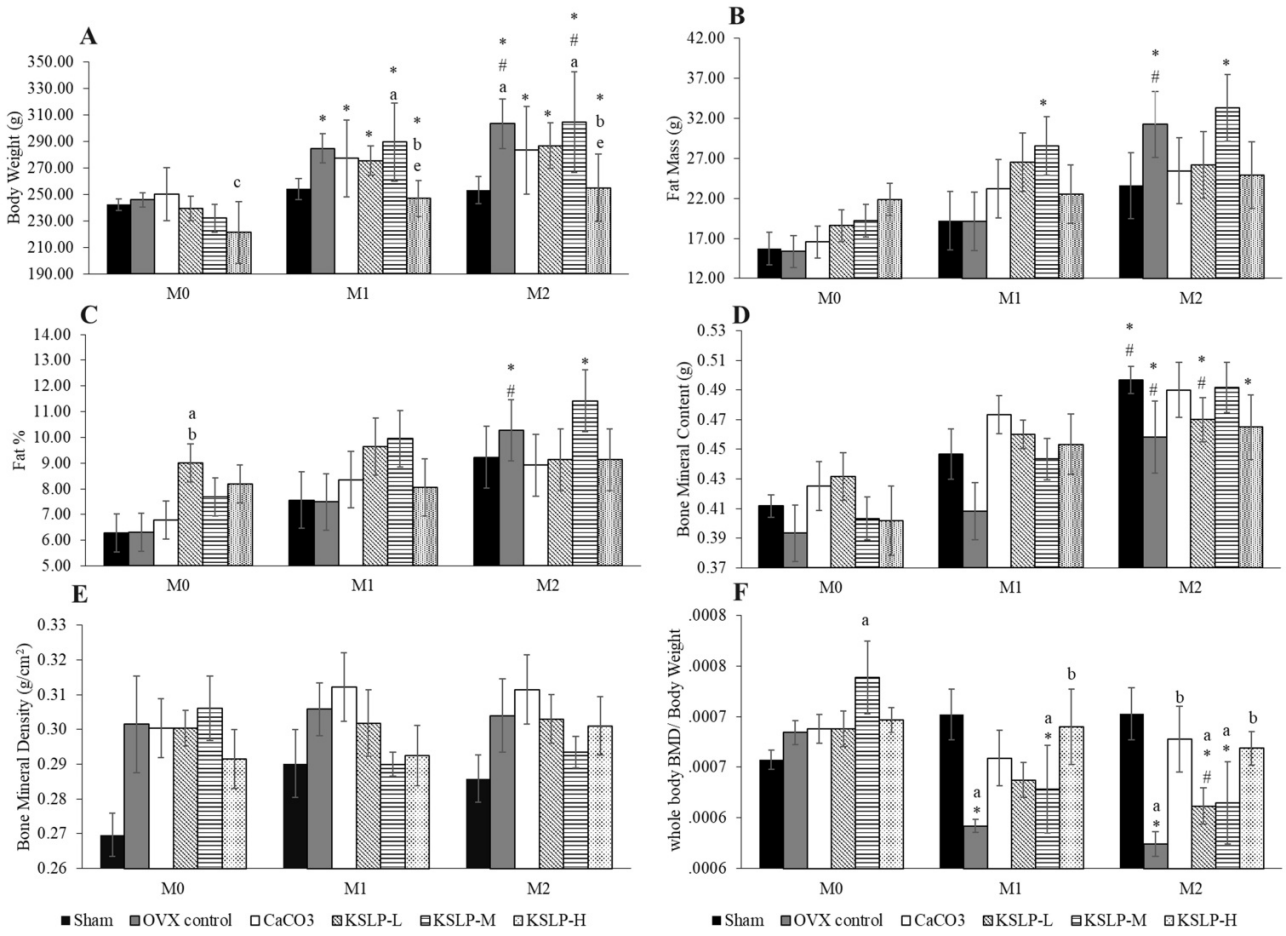
Body weight (**A**), fat mass (**B**), fat percentage (**C**), bone mineral content (**D**), bone mineral density (**E**) and whole-body bone mineral density/body weight (**F**) of each study group (n=6/group) during the experiment. The data are expressed as mean ± standard error mean. Mixed-design ANOVA is used to analyse the time and group differences. ^a^*p* < 0.05 vs sham, ^b^*p* < 0.05 vs OVX control, ^c^*p* < 0.05 vs CaCO_3_, ^e^*p* < 0.05 vs KSLP-M within the same month; ^*^p < 0.05 vs M0, ^#^*p* < 0.05 vs M1 within the same group. Abbreviations: CaCO_3_, calcium carbonate; KSLP-L, KSLP-low dose; KSLP-M, KSLP-medium dose; KSLP-H, KSLP-high dose; OVX, ovariectomised or negative; M0, month 0; M1, month 1; M2, month 2; %, percentage; BMD, bone mineral density.

All groups, except the sham, showed a significant increase in body weight with time. The body weight of OVX control and KSLP-M was significantly higher at month 1 and 2 compared to month 0 (p<0.001), and at month 2 compared to month 1 (OVX: p=0.024; KSLP-M: p=0.004). The body weight of KSLP-L (p<0.001 at month 1 and 2) and KSLP-H (month 1: p=0.008; month 2: p=0.004) at month 1 and 2 was significantly higher compared to month 0. The between-group comparison showed that body weight was significantly higher in the KSLP-M compared to the sham group (p=0.046), and decreased in the KSLP-H compared to the negative control (p=0.026) and KSLP-M group (p=0.008) at month 1. The body weight was significantly higher in the negative control (p=0.029) and KSLP-M (p=0.023) compared to the sham group at month 2. The KSLP-H group showed a significantly lower body weight compared to the negative control (p=0.039) and KSLP-M group (p=0.031) at month 2 (Figure 1A).

Fat mass increased significantly in the OVX control at month 2 compared to month 0 (p<0.001) and 1 (p<0.001). A similar increase was observed in the KSLP-M group at month 1 (p=0.025) and 2 (p=0.001) compared to month 0. No significant between-group difference was observed in fat mass at each time point (p>0.05) (Figure 1B). Similar increase in body fat percentage was observed in the OVX control (month 1 vs month 0: p=0.005; month 2 vs month 0: p=0.005), and in the KSLP-M group (month 2 vs month 0: p=0.009). At month 0, body fat percentage was higher in the KSLP-L group compared to the sham (p=0.023) and OVX control group (p=0.025) (Figure 1C).

Whole-body BMC increased significantly in the sham, OVX control and KSLP-M group at month 2 compared to month 0 (sham: p=0.004; OVX control: p=0.032; KSLP-M: p=0.003), and at month 2 compared to month 1 (sham: p=0.025; OVX control: p=0.025; KSLP-M: p=0.031). Whole-body BMC also increased in the CaCO_3_ (p=0.032) and KSLP-H group (p=0.038) at month 2 compared to month 0. However, no significant between-group differences in whole-body BMC were observed at each time point (p>0.05) (Figure 1D). All study groups showed no significant time-dependent changes and between-group differences in whole-body BMD (p>0.05) (Figure 1E).

Ovariectomy-induced weight gain could potentially confound the effects of oestrogen deficiency on BMD 19. Therefore, whole-body BMD values adjusted with body weight (BMD/body weight) were analysed. A time-dependent decrease of whole-body BMD/body weight was observed in the OVX control (month 2 vs month 0: p<0.001; month 1 vs month 0: p=0.001), KSLP-M (month 2 vs month 0: p<0.001; month 1 vs month 0: p<0.001) and KSLP-L (month 2 vs month 0: p=0.007; month 2 vs month 1: p=0.027). At month 0, whole BMD/body weight was higher in the KSLP-M group compared to the sham group (p=0.005). At month 1, whole BMD/body weight was significantly lower in the negative control (p=0.004) and KSLP-M group (p=0.047) compared to the sham group. At the same time point, whole BMD/body weight was significantly higher in the KSLP-H group compared to the OVX control (p=0.010). At month 2, whole BMD/body weight was significantly lower in the OVX control (p=0.002), KSLP-L (p=0.020) and KSLP-M (p=0.024) groups compared to the sham group. The whole BMD/body weight was higher in the CaCO_3_ group compared to OVX control at month 2 (p=0.009), while the KSLP-H group showed significantly higher whole BMD/body weight values compared to the OVX control at month 1 (p=0.010) and 2 (p=0.016) (Figure 1F).

### Bone remodelling markers

**Figure 2:**
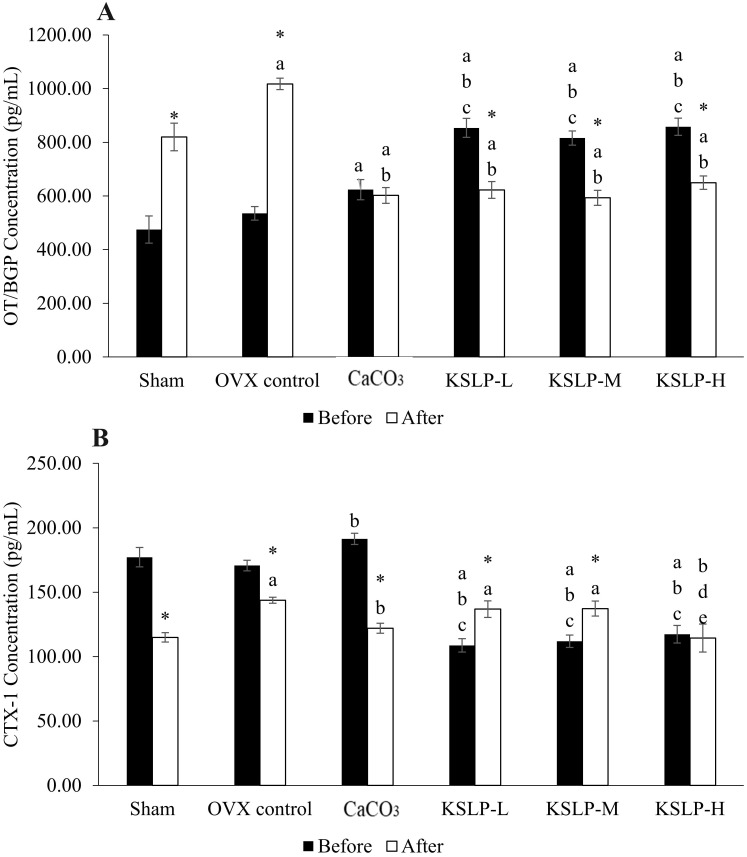
Osteocalcin (**A**) and CTX-1 (**B**) concentration of each study group (n=6/group) before and after the experiment. The data are expressed as mean ± standard error mean. Mixed-design ANOVA is used to analyse the time and group differences. ^a^p < 0.05 vs sham; ^b^p < 0.05 vs OVX control; ^c^p < 0.05 vs CaCO_3_; ^d^p < 0.05 vs KSLP-L; ^e^p < 0.05 vs KSLP-M with the same time; ^*^p < 0.05 vs before treatment in same group. Abbreviations: CaCO_3_, calcium carbonate; KSLP-L, KSLP-low dose; KSLP-M, KSLP-medium dose; KSLP-H, KSLP-high dose; OT/BGP, Osteocalcin/Bone Gla protein; OVX, ovariectomised or negative; CTX-1, Type I collagen cross-linked c-telopeptide.

Serum osteocalcin increased significantly across the treatment period in the sham (p<0.001) and OVX control (p<0.001), but decreased significantly in the KSLP-L (p<0.001), -M (p<0.001) and -H group (p<0.001). The CaCO_3_ group did not show significant time-dependent changes in serum osteocalcin level (p=0.555) (Figure 2A). Between-group analysis before treatment showed that the CaCO_3_ group had a higher serum osteocalcin level than the sham group (p=0.006). The KSLP-L, -M and -H group showed significantly higher osteocalcin level compared to the sham, OVX control and CaCO_3_ group before treatment (p<0.001 for all). After treatment, serum osteocalcin level was significantly higher in the negative control than the sham, KSLP-L, -M, -H and CaCO_3_ group (p<0.001 for all). All supplemented groups also had a higher osteocalcin level compared to the sham and OVX control group (p<0.001 for all) (Figure 2A).

Serum CTX-1 level decreased significantly in the sham (p<0.001), negative control (p=0.002) and CaCO_3_ (p<0.001) group but decreased in the KSLP-L (p=0.001) and -M (p=0.003) groups across the study period (Figure 2B). At month 0, CTX-1 was higher in the CaCO_3_ group compared to the OVX control group (p=0.014), while it was lower in the KSLP-L, -M and -H group compared to the sham, OVX control and calcium groups (p<0.001 for all). After treatment, CTX-1 concentration was significantly higher in the OVX control (p=0.002), KSLP-L (p=0.017) and -M group (p=0.015) compared to the sham group, while it was significantly lower in the calcium group compared to the OVX control (p=0.018). At the same time, CTX-1 was significantly lower in the KSLP-H group compared to the OVX control (p=0.002), KSLP-L (p=0.015), and -M (p=0.013) group (Figure 2B).

### Bone histomorphometry

**Figure 3:**
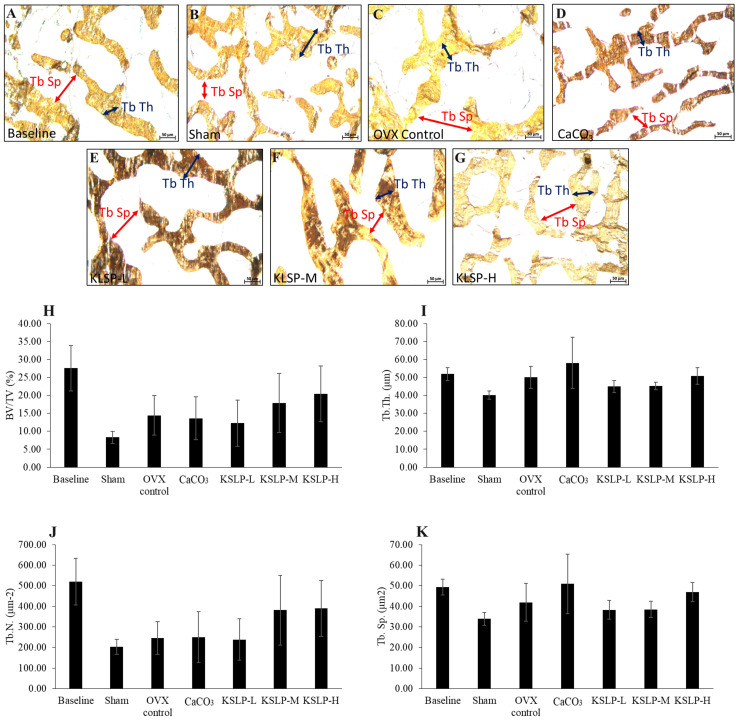
Micrograph of femur sections (100 × magnification) stained using the von Kossa method/silver nitrate (**A-G**) for each group (n=6/group). The structural histomorphometric indices of the femur evaluated are BV/TV (**H**), Tb.Th (**I**), Tb.N (**J**), and Tb.Sp (**K**). The quantitative data are expressed as mean ± standard error mean. The differences between groups are evaluated by one-way ANOVA with Tukey's post hoc test. Abbreviations: CaCO_3_, calcium carbonate; KSLP-L, KSLP-low dose; KSLP-M, KSLP-medium dose; KSLP-H, KSLP-high dose; OVX, ovariectomised or negative; BV/TV, bone volume/total volume; Tb.Th., trabecular bone thickness; Tb.N, trabecular bone number; Tb.Sp., trabecular bone separation

**Figure 4:**
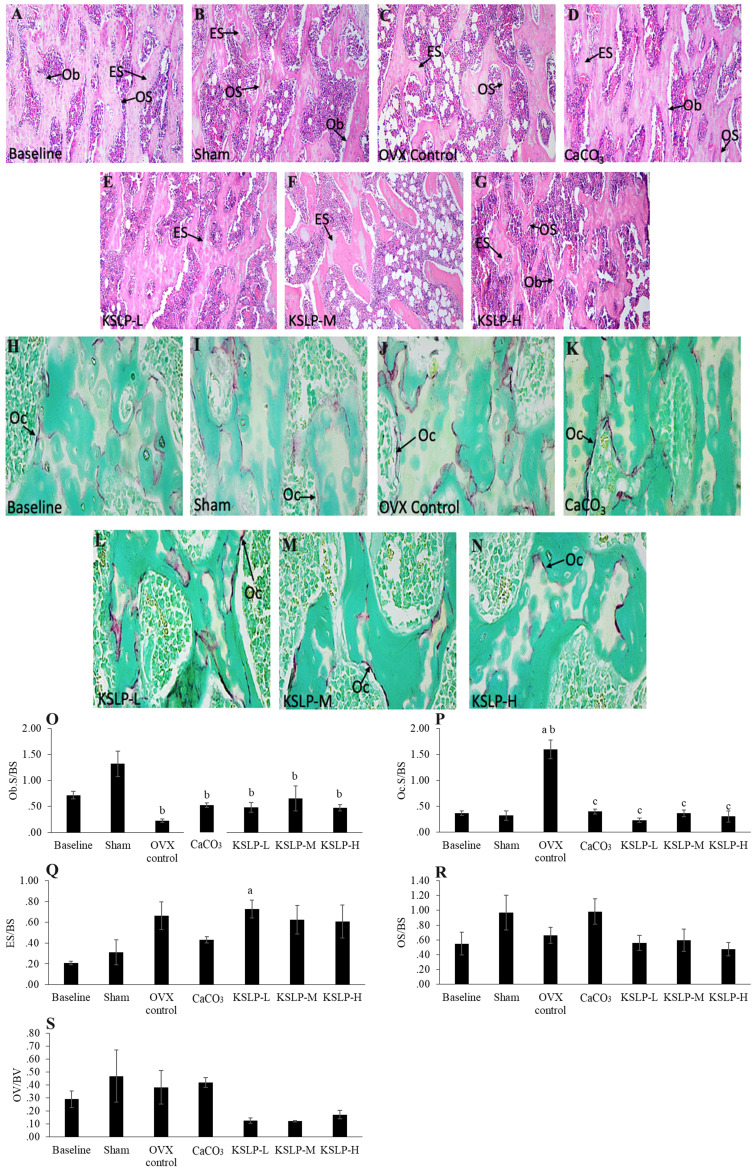
Micrograph of H & E- (**A-G**) (100 × magnification) and TRAP-stained (**H-N**) (100 × magnification) femur sections for each study group (n=6/group). Static histomorphometric indices of femur bone evaluated are Ob.S/BS (**O**), Oc.S/BS (**P**), ES/BS (**Q**), OS/BS (**R**) and OV/BV (**S**). The data are expressed as mean ± standard error mean. The differences between groups are evaluated by one-way ANOVA with Tukey's post hoc test.^ a^p < 0.05 vs baseline; ^b^p < 0.05 vs sham; ^c^p < 0.05 vs OVX control. Abbreviations: CaCO_3_, calcium carbonate; KSLP-L, KSLP-low dose; KSLP-M, KSLP-medium dose; KSLP-H, KSLP-high dose; Ob.S/BS, osteoblast surface; Oc.S/BS, osteoclast surface; OVX, ovariectomised or negative; ES/BS, eroded surface; OS/BS, osteoid surface; OV/BS, osteoid volume; Ob, osteoclast; Oc, osteoclast; ES, eroded surface; OS, osteoid

Bone structural histomorphometric analysis showed that BV/TV, Tb.Th, Tb.N and Tb.Sp did not differ significantly among the study groups (p>0.05) (Figure 3). For bone cellular histomorphometric indices, OS/BS and OV/BV did not differ significantly among the study groups (p>0.05). However, Ob.S/BS of OVX control, CaCO_3_, KSLP-L, -M and -H groups was significantly lower compared to the sham group (OVX control: p<0.001; CaCO_3_: p=0.005; KSLP-L: p=0.003; KSLP-M: p=0.028 and KSLP-H: p=0.003). Oc.S/BS of OVX control was significantly higher compared to the baseline and sham groups (p<0.001 for all). Oc.S/BS was significantly lowered in CaCO_3_, KSLP-L, -M and -H groups compared to the negative control (p<0.001). ES/BS was significantly higher in the KSLP-L group compared to the baseline group (p=0<0.001) (Figure 4).

**Figure 5:**
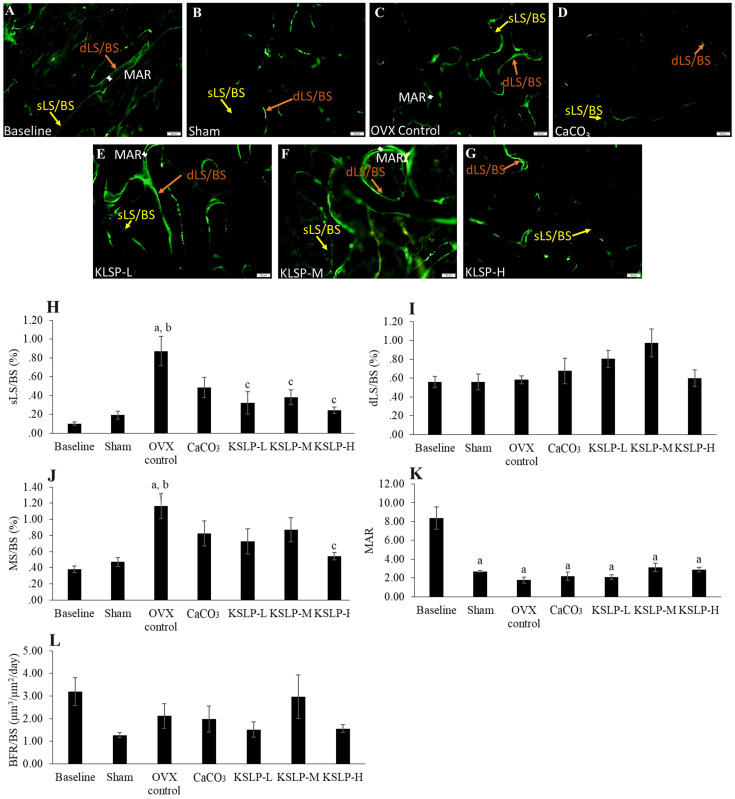
** Micrograph of calcein-labelled femur sections (A-G)** (200 × magnification) for each study group (n=6/group); Dynamic histomorphometric indices of femur bone evaluated are sLS/BS (**H**), dLS/BS (**I**), MS/BS (**J**), MAR (**K**) and BFR (**L**). The data are expressed as mean ± standard error mean. One-way ANOVA with Tukey's post hoc test is used to compare the difference between groups. ^a^p < 0.05 vs baseline; ^b^p < 0.05 vs sham; ^c^p < 0.05 vs OVX control. Abbreviations: CaCO_3_, calcium carbonate; KSLP-L, KSLP-low dose; KSLP-M, KSLP-medium dose; KSLP-H, KSLP-high dose; OVX, ovariectomised or negative; sLS/BS, single-labelled surface; dLS/BS, doubled-labelled surface; MS/BS, mineralizing surface; MAR, mineral apposition rate; BFR, bone formation rate

Bone dynamic histomorphometry revealed that sLS/BS was significantly increased in the OVX control compared to the baseline (p<0.001) and sham (p<0.001). KSLP treatment at all doses lowered sLS/BS values compared to the OVX control (KSLP-L: p=0.003; KSLP-M: p=0.010; KSLP-H: p=0.001). No significant difference in dSL/BS was detected among all the study groups (p>0.05). MS/BS was significantly increased in the OVX control than the baseline (p=0.001) and sham (p=0.005), while the increase was prevented with high-dose KSLP (p=0.014). MAR was significantly decreased in the sham (p<0.001), OVX control (p<0.001), CaCO_3_ (p<0.001) and KSLP (KSLP-L: p<0.01; KSLP-M: p<0.001; KSLP-H: p<0.001) compared to the baseline. No significant difference was detected in BFR/BS among all the study groups (p>0.05) (Figure 5).

### Biomechanical strength

**Figure 6:**
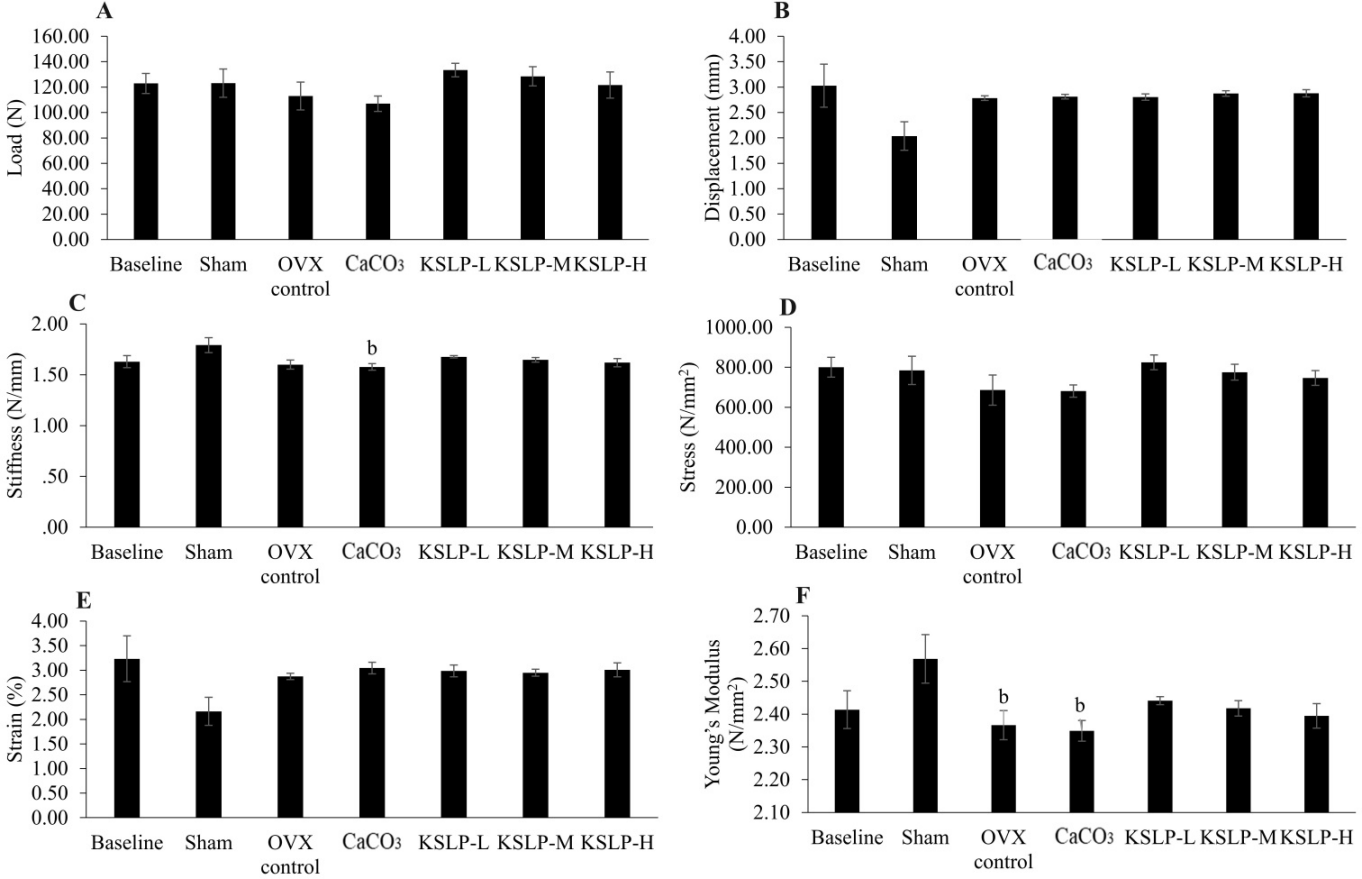
** Bone mechanical strength indices of each group (n=6/rats) evaluated based on three-point-bending test.** The parameters generated are load (**A**), displacement (**B**), stiffness (**C**), stress (**D**), strain (**E**) and Young's modulus (**F**). The data are expressed as mean ± standard error mean. One-way ANOVA with Tukey's post hoc test is used to compare the difference between groups. ^b^p < 0.05 vs sham. Abbreviations: CaCO_3_, calcium carbonate; KSLP-L, KSLP-low dose; KSLP-M, KSLP-medium dose; KSLP-H, KSLP-high dose; OVX, ovariectomised or negative.

No significant differences in load, displacement, stress, and strain were observed among the study groups (p>0.05). Stiffness in the CaCO_3_ group was significantly decreased compared to the negative control (p=0.028) while Young's modulus was significantly decreased in the OVX control (p=0.041) and CaCO_3_ compared to the sham (p=0.020) (Figure 6).

## Discussion

The current study found that OVX-induced oestrogen deficiency resulted in a high bone remodelling state, as evidenced by increased osteocalcin and CTX-1 levels, as well as higher sLS/BS and MS/BS ratios. This might be induced by increased Oc.S/BS and decreased Ob.S/BS and bone formation activities (MAR). As a result, the OVX rats had lower whole-body BMD/body weight and Young's modulus values, indicating a reduction in bone mass and strength. Other indices of biomechanical strength, static and structural histomorphometric indices did not change in response to OVX, possibly because they take longer to manifest. KSLP treatment significantly suppressed the high bone remodelling event and Oc.S/BS, leading to an increase in whole-body BMD/body weight. OVX also caused an increase in body fat but this was prevented by KSLP treatment.

In rats, removing ovarian hormones via ovariectomy increases daily food consumption as well as body mass and body fat mass 20. Ovariectomy increases the activity of lipoprotein lipase in adipose tissue involved in the hydrolysis, absorption and storage of triglycerides in adipocytes, thereby boosting fat mass accumulation 21. In the present study, OVX rats showed increased body weight over time compared to the sham as observed in other studies 22, 23. The increase in body weight agreed with the increase in fat mass and fat percentage of the OVX rats. In another study by Mohamad et al. 19, ovariectomy caused a significant increase in body weight without the accompanying increase in fat mass after 8 weeks. In contrast, body mass, fat mass and adipose tissue (subcutaneous and intra-abdominal) increased in OVX rats after 41 days in another study 24. The difference could arise from the standard rat chow provided to the rats in individual studies. KSLP supplementation did not produce significant effects on the fat mass and fat percentage in the rats. Only KSLP at the highest dose significantly suppressed ovariectomy-induced body weight gain. However, the exact weight reduction mechanism of KSLP is unclear at this moment.

BMC represents the total mineral content of the bone 25. Elesawy et al. 26 found a decrease in BMC in OVX rats after 16 weeks. In the current study, OVX did not affect BMC values after 8 weeks, implying that more time is required for significant changes to develop. In addition, BMC was not affected by KSLP supplementation. BMD represents mineral content divided by bone area 25. Previous studies by Georgieva et al. 27 and Mohamad et al. 19 showed that OVX did not affect BMD 2-3 months post ovariectomy. Similarly, OVX did not affect BMD after 8 weeks in this study, implying that more time may be required for the changes in BMD to become evident. Increased mechanical loading due to higher body weight could mask the negative effects of oestrogen deficiency on bone. In this study, a decrease in BMD values normalised by body weight was detected in the OVX group. High-dose KSLP supplementation increased whole-body BMD adjusted for body weight compared to the negative control. The dried root extract of one of the ingredients of KSLP, *Rehmannia glutinosa*, has been reported by Lim and Kim 10 to prevent BMD decrease in OVX rats.

In the intact growing female rats of this study, time-dependent increase in osteocalcin and decrease in CTX-1 were observed probably due to continuous bone growth. This observation is similar to previous studies 28, 29. Oestrogen deficiency results in rapid bone loss due to an increase in bone turnover and an imbalance between bone resorption and formation 30. In this study, this phenomenon was evidenced by the concurrent increase in osteocalcin (bone formation marker) and CTX-1 (bone resorption marker). The results were similar to previous studies which reported higher CTX-1 and osteocalcin in OVX animals 31, 32. In the present study, all doses of KSLP significantly reduced serum osteocalcin but only high-dose KSLP reduced CTX-1 levels, suggesting suppression of high bone remodelling associated with OVX. Lim and Kim 10 reported that the dried root extract of *Rehmannia glutinosa* suppressed bone remodelling in OVX rats, as evidenced by decreased serum alkaline phosphatase levels. On the other hand, calcium carbonate alone failed to suppress high CTX-1 levels in OVX rats. A similar observation was obtained in female rats castrated surgically and in male rats castrated chemically 29, 33.

Bone histomorphometry allows researchers to examine bone micro-architecture, bone cellular changes and formation/resorption activities and bone remodelling quantitatively in a two-dimensional manner 34, 35. Bone structural histomorphometric parameters assess bone volumetric and microstructural changes resulted from bone remodelling process 34. Previous studies revealed that ovariectomy decreased BV/TV and Tb.N., and increased Tb.Sp. after 6 to 8 weeks 36, 37. However, the present study revealed ovariectomy did not alter bone structural metrics, implying that high bone remodelling has yet to impair bone microstructure. KSLP supplementation had no discernible effect on bone microstructure.

Bone cellular histomorphometry assesses the number of osteoblasts and osteoclasts, the amount of unmineralized bone (as osteoid), and the extent of bone resorption (as eroded surface) 34. According to studies by Mohamad et al. 38 and Mohamed et al. 37, OVX did not affect ES/BS and OS/BS but reduced OV/BV. In the present study, there were no OVX-induced changes to ES/BS, OS/BS and OV/BV probably due to the large within-group variance. However, Ob.S./BS was significantly reduced while Oc.S/BS was significantly increased by OVX, suggesting a disruption in the balance between bone resorption and formation 39. Similarly, KSLP supplementation did not affect Ob.S/BS, ES/BS, OS/BS and OV/BV compared to the negative control. However, high-dose KSLP supplementation reduced Oc.S/BS as evidenced by a decrease in TRAP-stained cells, suggesting reduced TRAP activity/osteoclast differentiation. This was in line with the study by Huang et al. 15, wherein treatment with Ophiopogonin D, a natural product extracted from *Ophiopogonis radix* (an ingredient of KSLP), reduced TRAP activity in RAW264.7 cells and OVX-induced osteopenic rodents.

Bone dynamic histomorphometry examines skeletal mineralisation activities across a period 34. In the current study, increased active mineralisation indicated by MS/BS was observed in the OVX control group, which could be a result of increased bone remodelling. However, the increased sLS/BS and reduced MAR in OVX rats indicated diminished new mineral apposition on the bone surface 34. These findings were also reported by Fernandes and Gomes 40, whereby new bone deposition was reduced with oestrogen deficiency. Although all doses of KSLP reduced sLS/BS, only the high dose was able to lower MS/BS, suggesting that it may have some potential in preventing excessive bone remodelling. In this investigation, all doses of KSLP exerted no effect on dLS/BS, MAR, or BFR/BS, implying that they were ineffective in enhancing bone formation rate.

The most common mechanical test used to characterise the biomechanical properties of long bones is the three-point bending test 41. It is a simple, affordable, and reliable method of assessing bone health because it accurately reflects bone quality 42. A previous study showed that 4 months after ovariectomy, load, stiffness, stress, and Young's modulus of elasticity were significantly reduced in female rats, but strain and displacement were not significantly altered 19. The present study revealed that ovariectomy has no effect on both intrinsic and extrinsic biomechanical properties of the bone, but it reduced Young's modulus after 2 months suggesting a high tendency for the femur to be deformed elastically when force is applied to it 43. In comparison to the OVX control, KSLP supplementation did not alter the skeletal intrinsic and extrinsic properties, implying that biomechanical indices need a longer time to show changes following ovariectomy and treatment.

KLSP did not display obvious dose-dependent effects in most skeletal parameters measured in this study, indicating the lowest dose was effective in preventing bone loss. The effectiveness of CaCO_3_ in preventing bone loss has been demonstrated in both castrated male and female rats 19, 33, 38, 44. However, its efficacy in protecting against bone loss was lower compared to KSLP.

Some limitations of this study should be addressed. Due to the unavailability of the micro-computed tomography machine, the skeletal microstructure was quantified using two-dimensional histomorphometry only. Osteoblast identification was performed using cell morphology rather than alkaline phosphatase staining. Mechanistic investigations were not performed to examine the bone protective mechanism of KSLP. KSLP constituents have been reported to possess antioxidant and anti-inflammatory activities 15, 45-49. It has been shown to reduce lipid peroxidation in the brain of D-galactose-induced aged rats 50. It has been reported that oxidative stress and inflammation stimulate osteoclast differentiation, resulting in increased bone resorption 51, 52, implying that the reduction in Oc.S/BS in the current study could be due to the antioxidant and anti-inflammatory activity of KSLP. For bone remodelling markers, significant differences between KSLP groups and other groups prior to treatment were noted. This could be due to physiological variation among the rats, which could not be adjusted before the experiment started. This is because the biochemical assays were conducted after the rats were sacrificed.

## Conclusion

KSLP prevented bone loss in oestrogen deficient mice by normalising bone remodelling at all three doses. However, due to the limitations of the current model, which exhibited marginal deterioration in bone structure and biomechanical strength 8 weeks after ovariectomy, the effects of KSLP on these aspects cannot be evaluated. We suggest a more in-depth study with a longer trial period and mechanistic examination to establish the skeletal protective effects of KSLP.

## References

[1] Mohamad NV, Ima-Nirwana S, Chin KY (2020). Are Oxidative Stress and Inflammation Mediators of Bone Loss Due to Estrogen Deficiency? A Review of Current Evidence. Endocr Metab Immune Disord Drug Targets.

[2] Eastell R, O'Neill TW, Hofbauer LC, Langdahl B, Reid IR, Gold DT (2016). Postmenopausal osteoporosis. Nat Rev Dis Primers.

[3] Lorentzon M, Johansson H, Harvey NC, Liu E, Vandenput L, McCloskey EV (2022). Osteoporosis and fractures in women: the burden of disease. Climacteric.

[4] Kanis JAA, Cooper C, Rizzoli R, Reginster JYY, Clinical obotSABotESf, Economic Aspects of O (2019). European guidance for the diagnosis and management of osteoporosis in postmenopausal women. Osteoporos Int.

[5] Black DM, Rosen CJ (2016). Clinical Practice. Postmenopausal Osteoporosis. N Engl J Med.

[6] Suvarna V, Sarkar M, Chaubey P, Khan T, Sherje A, Patel K (2018). Bone Health and Natural Products- An Insight. Front Pharmacol.

[7] Mukwaya E, Xu F, Wong M-S, Zhang Y (2014). Chinese herbal medicine for bone health. Pharm Biol.

[8] He JB, Chen MH, Lin DK (2017). New insights into the tonifying kidney-yin herbs and formulas for the treatment of osteoporosis. Arch Osteoporos.

[9] Gong S, Ye T, Wang M, Wang M, Li Y, Ma L (2020). Traditional Chinese Medicine Formula Kang Shuai Lao Pian Improves Obesity, Gut Dysbiosis, and Fecal Metabolic Disorders in High-Fat Diet-Fed Mice. Front Pharmacol.

[10] Lim DW, Kim YT (2013). Dried root of Rehmannia glutinosa prevents bone loss in ovariectomized rats. Molecules.

[11] Lei L, Chen Y, Ou L, Xu Y, Yu X (2017). Aqueous root extract of Asparagus cochinchinensis (Lour.) Merr. Has antioxidant activity in D-galactose-induced aging mice. BMC Complement Altern Med.

[12] Kim MH, Lee JE, Lee JS, Yang WM (2017). Improvement of osteoporosis by Lycium chinense administration in ovariectomized mice. J Chin Med Assoc.

[13] Kepekci AH, Ergul Z, Gultekin A, Karaoz E (2019). The effect of Korean red ginseng on mesenchymal stem cells from healthy and osteoporotic human bone marrow. Int J Physiol Pathophysiol Pharmacol.

[14] Hwang Y-HH, Jang S-AA, Lee A, Kim T, Ha H (2020). Poria cocos ameliorates bone loss in ovariectomized mice and inhibits osteoclastogenesis *in vitro*. Nutrients.

[15] Huang Q, Gao B, Wang L, Zhang H-YY, Li X-JJ, Shi J (2015). Ophiopogonin D: A new herbal agent against osteoporosis. Bone.

[16] Li W, Hong Z, Gong S, Qu H, Qian J, Chen X (2018). The Effects of Traditional Chinese Medicine KSLP on Adipose-derived stem cells in Aged Mice. Regional Conference on Non-Communicable Diseases. Selangor.

[17] Reagan-Shaw S, Nihal M, Ahmad N (2008). Dose translation from animal to human studies revisited. Faseb J.

[18] Subramaniam S, Mohamad NV, Chan CY, Soelaiman IN, Chin KY (2020). Calculating *In-vivo* Short-term Precision Error of Dual-Energy X-ray Absorptiometry in Human and Animal: A Technical Report. Medicine & Health.

[19] Mohamad N-VV, Ima-Nirwana S, Chin K-YY (2021). Therapeutic potential of annatto tocotrienol with self-emulsifying drug delivery system in a rat model of postmenopausal bone loss. Biomed Pharmacother.

[20] Chen Y, Heiman ML (2001). Increased weight gain after ovariectomy is not a consequence of leptin resistance. Am J Physiol Endocrinol Metab.

[21] Toth MJMJ, Poehlman ETET, Matthews DEDE, Tchernof A, MacCoss MJMJ (2001). Effects of estradiol and progesterone on body composition, protein synthesis, and lipoprotein lipase in rats. Am J Physiol Endocrinol Metab.

[22] Muhammad N, Razali S, Shuid AN, Mohamed N, Soelaiman IN (2013). Comparing the Effects of Tocotrienol-rich Fraction, Calcium and Estrogen on Bone Metabolism in Ovariectomized Rats. Sains Malay.

[23] Muhammad N, Luke DA, Shuid AN, Mohamed N, Soelaiman IN (2013). Tocotrienol supplementation in postmenopausal osteoporosis: evidence from a laboratory study. Clinics (Sao Paulo).

[24] Gloy V, Langhans W, Hillebrand JJ, Geary N, Asarian L (2011). Ovariectomy and overeating palatable, energy-dense food increase subcutaneous adipose tissue more than intra-abdominal adipose tissue in rats. Biol Sex Differ.

[25] Friedman AW (2006). Important determinants of bone strength: beyond bone mineral density. J Clin Rheumatol.

[26] Elesawy BH, Sakr HF, Abbas AM (2021). Synergistic Protective Effects of Resveratrol and Estradiol on Estrogen Deficiency-Induced Osteoporosis Through Attenuating RANK Pathway. Int J Pharmacol.

[27] Georgieva A, Eftimov M, Todorova M, Kuzmanova V, Kuzmanov A, Kuzmanov K (2021). Effects of ovariectomy-induced estrogen deficit on rat behaviour, lipid metabolism, inflammation, bone mineral density, and turnover. Folia Medica.

[28] Aktifanus AT, Shuid AN, A (2012). Rashid NH, Tam HL, Chua YL, M. Saat N, et al. Comparison of the Effects of Tocotrienol and Estrogen on the Bone Markers and Dynamic Changes in Postmenopausal Osteoporosis Rat Model. Asian J Anim Vet Adv.

[29] Soelaiman IN, Ming W, Abu Bakar R, Hashnan NA, Mohd Ali H, Mohamed N (2012). Palm tocotrienol supplementation enhanced bone formation in oestrogen-deficient rats. Int J Endocrinol.

[30] Cheng C-HH, Chen L-RR, Chen K-HH (2022). Osteoporosis Due to Hormone Imbalance: An Overview of the Effects of Estrogen Deficiency and Glucocorticoid Overuse on Bone Turnover. Int J Mol Sci.

[31] Yoon K-H, Cho D-C, Yu S-H, Kim K-T, Jeon Y, Sung J-K (2012). The Change of Bone Metabolism in Ovariectomized Rats: Analyses of MicroCT Scan and Biochemical Markers of Bone Turnover. J Korean Neurosurg Soc.

[32] Kim T-H, Jung JW, Ha BG, Hong JM, Park EK, Kim H-J (2011). The effects of luteolin on osteoclast differentiation, function *in vitro* and ovariectomy-induced bone loss. J Nutr Biochem.

[33] Mohamad NV, Soelaiman IN, Chin KY (2018). Effects of tocotrienol from Bixa orellana (annatto) on bone histomorphometry in a male osteoporosis model induced by buserelin. Biomed Pharmacother.

[34] Kulak CAM, Dempster DW (2010). Bone histomorphometry: a concise review for endocrinologists and clinicians. Arq Bras Endocrinol Metabol.

[35] Malhan D, Muelke M, Rosch S, Schaefer AB, Merboth F, Weisweiler D (2018). An Optimized Approach to Perform Bone Histomorphometry. Front Endocrinol.

[36] da Paz LH, de Falco V, Teng NC, dos Reis LM, Pereira RM, Jorgetti V (2001). Effect of 17beta-estradiol or alendronate on the bone densitometry, bone histomorphometry and bone metabolism of ovariectomized rats. Braz J Med Biol Res.

[37] Mohamed N, Sahhugi Z, Ramli ESM, Muhammad N (2013). The effects of Cosmos caudatus (ulam raja) on dynamic and cellular bone histomorphometry in ovariectomized rats. BMC Res Notes.

[38] Mohamad N-VV, Ima-Nirwana S, Chin K-YY (2021). Self-emulsified annatto tocotrienol improves bone histomorphometric parameters in a rat model of oestrogen deficiency through suppression of skeletal sclerostin level and RANKL/OPG ratio. Int J Med Sci.

[39] Chen X, Wang Z, Duan N, Zhu G, Schwarz EM, Xie C (2018). Osteoblast-osteoclast interactions. Connect Tissue Res.

[40] Fernandes MH, Gomes PS (2016). Bone Cells Dynamics during Peri-Implantitis: a Theoretical Analysis. J Oral Maxillofac Res.

[41] Osuna LGG, Soares CJ, Vilela ABF, Irie MS, Versluis A, Soares PBF (2020). Influence of bone defect position and span in 3-point bending tests: experimental and finite element analysis. Braz Oral Res.

[42] Oksztulska-Kolanek E, Znorko B, Michałowska M, Pawlak K (2016). The Biomechanical Testing for the Assessment of Bone Quality in an Experimental Model of Chronic Kidney Disease. Nephron.

[43] Fathilah SN, Abdullah S, Mohamed N, Shuid AN (2012). Labisia pumila prevents complications of osteoporosis by increasing bone strength in a rat model of postmenopausal osteoporosis. Evid Based Complement Alternat Med.

[44] Mohamad NV, Ima-Nirwana S, Chin KY (2018). Effect of tocotrienol from bixa orellana (Annatto) on bone microstructure, calcium content, and biomechanical strength in a model of male osteoporosis induced by buserelin. Drug Des Devel Ther.

[45] Ahn M, Park JS, Chae S, Kim S, Moon C, Hyun JW (2014). Hepatoprotective effects of Lycium chinense Miller fruit and its constituent betaine in CCl4-induced hepatic damage in rats. Acta Histochem.

[46] Baek G-HH, Jang Y-SS, Jeong S-II, Cha J, Joo M, Shin S-WW (2012). Rehmannia glutinosa suppresses inflammatory responses elicited by advanced glycation end products. Inflammation.

[47] Han SY, Kim J, Kim E, Kim SH, Seo DB, Kim JH (2018). AKT-targeted anti-inflammatory activity of Panax ginseng calyx ethanolic extract. J Ginseng Res.

[48] Jeong J-WW, Lee HHH, Han MHH, Kim G-YY, Hong SHH, Park C (2014). Ethanol extract of Poria cocos reduces the production of inflammatory mediators by suppressing the NF-kappaB signaling pathway in lipopolysaccharide-stimulated RAW 264.7 macrophages. BMC Complement Altern Med.

[49] Lee HA, Koh EK, Sung JE, Kim JE, Song SH, Kim DS (2017). Ethyl acetate extract from Asparagus cochinchinensis exerts anti-inflammatory effects in LPS-stimulated RAW264.7 macrophage cells by regulating COX-2/iNOS, inflammatory cytokine expression, MAP kinase pathways, the cell cycle and anti-oxidant activity. Mol Med Rep.

[50] Tao TP (2009). Effects of KSLP on learning and memory and free radical metabolism in aging rats. J Zhejiang Univ Tradit Chin Med.

[51] Galliera E, Massaccesi L, Banfi G, De Vecchi E, Ragone V, Corsi Romanelli MM (2021). Effect of Oxidative Stress on Bone Remodeling in Periprosthetic Osteolysis. Clin Rev Bone Miner Metab.

[52] Luo G, Li F, Li X, Wang ZG, Zhang B (2018). TNFalpha and RANKL promote osteoclastogenesis by upregulating RANK via the NFkappaB pathway. Mol Med Rep.

